# Breast Mass as an Atypical Presentation of Non-Hodgkin Lymphoma

**DOI:** 10.7759/cureus.97574

**Published:** 2025-11-23

**Authors:** Filipa Figueiredo, Teresa Valido, Carolina Chumbo, Garcieth Gomes, Marta Rocha

**Affiliations:** 1 Internal Medicine, Hospital Professor Doutor Fernando Fonseca, Amadora, PRT

**Keywords:** breast lymphoma, breast mass, diffuse large b-cell lymphoma, lymphoproliferative disorders, multidisciplinary approach

## Abstract

Primary breast lymphoma (PBL) is a rare malignant entity, accounting for less than 0.5% of all breast malignancies. It must be differentiated from conventional breast carcinoma, as the therapeutic approach and outcomes differ. We present the case of an 86-year-old woman diagnosed with diffuse large B-cell non-Hodgkin lymphoma (NHL), non-germinal centre subtyp*e*, of the breast, whose only symptom was a palpable breast mass. The patient declined all proposed therapies. This case highlights the importance of considering breast lymphoma in the differential diagnosis of breast masses and emphasises the need for a multidisciplinary approach to ensure accurate diagnosis and appropriate management.

## Introduction

Primary breast lymphoma (PBL) is a rare clinical entity, first described in 1959 by Dobrotina [1,2]. It is defined as a malignant lymphoma occurring primarily in the breast without prior evidence of lymphoma elsewhere [2]. PBL originates from lymphocytes [3], although its aetiology is not clearly understood [1].

Most cases are of the non-Hodgkin B-cell type, with diffuse large B-cell lymphoma (DLBCL) being the predominant subtype, representing approximately half of all PBLs [4]. Less common subtypes include follicular lymphoma, mucosa-associated lymphoid tissue (MALT) lymphoma, and Burkitt lymphoma [2]. Clinically, PBL usually presents as a solitary, palpable nodule [4], with imaging features that may mimic breast carcinoma [1,3,5]. Accurate differentiation from carcinoma is essential, as treatment approaches differ and unnecessary mastectomies can be avoided. Diagnosis is confirmed by biopsy, and treatment typically involves a combination of chemotherapy and radiotherapy [3].

PBL accounts for approximately 0.4-0.5% of all breast malignancies [6]. According to the WHO 5th Edition of Haematolymphoid Tumours, PBL is classified among B-cell non-Hodgkin lymphomas (NHLs), with DLBCL remaining the predominant subtype, reflecting updated definitions and diagnostic criteria [7]. This case highlights a rare presentation in an elderly, asymptomatic patient with an incidental breast mass, illustrating a diagnostic challenge in this population.

## Case presentation

**Figure 1:**
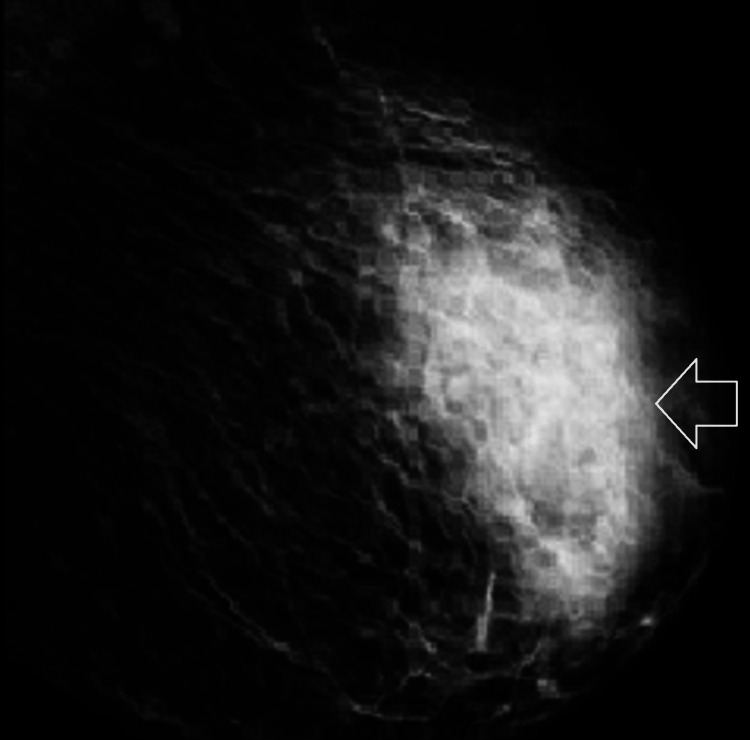
Mammography revealing a radio-opaque lesion with irregular margins in the upper outer quadrant of the left breast

**Figure 2:**
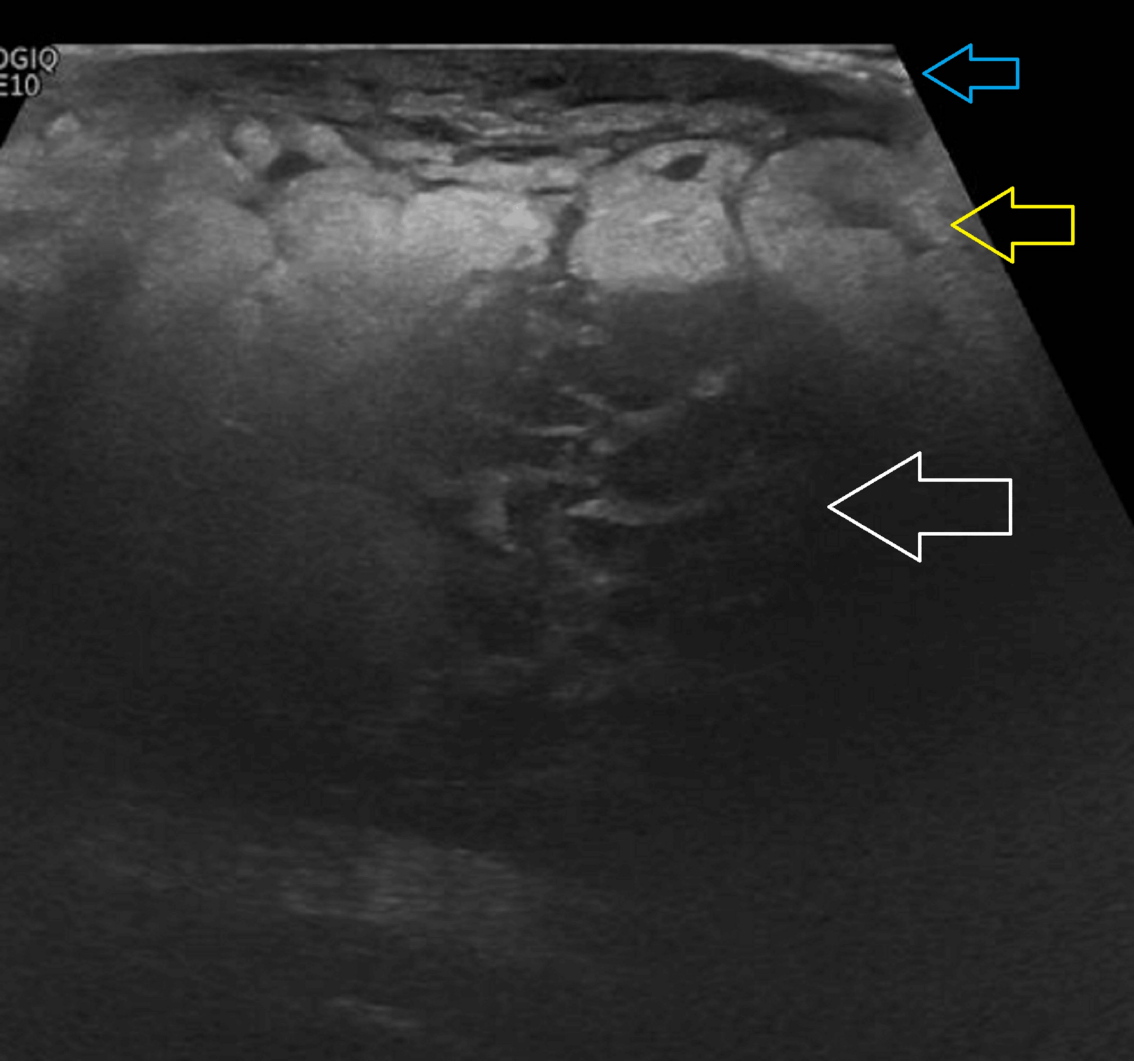
Ultrasound of the breast This image demonstrates increased skin thickness (blue arrow), underlying subcutaneous edema (yellow arrow), and diffuse infiltration of the breast tissue (white arrow), consistent with lymphoma involvement

We present the case of an 86-year-old woman with a medical history of hypertension and atrial fibrillation. She was evaluated in the emergency department after an accidental fall at home, without loss of consciousness, resulting in a traumatic brain injury. A cranial computed tomography (CT) revealed a right frontotemporal epicranial haematoma and right periorbital soft tissue injury. During physical examination, an incidental palpable mass was found in the left breast, difficult to delineate, solid, and immobile. No other visible breast alterations were noted, such as skin retraction, erythema, or nipple discharge. The patient was subsequently admitted for monitoring and further investigation of the suspicious breast lesion. To further characterise the lesion, mammography (Figure 1) and ultrasound (Figure 2) were performed.

**Figure 3:**
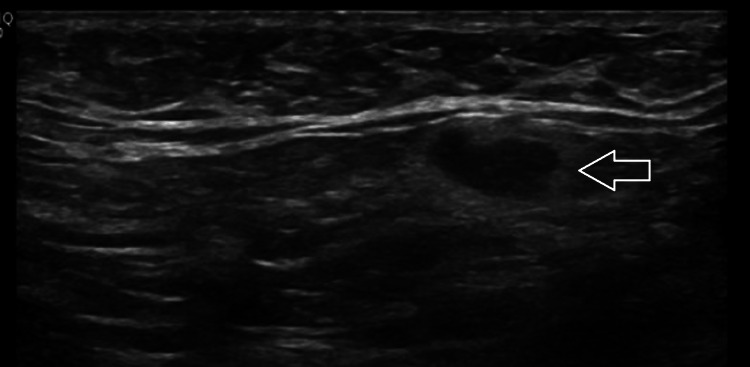
Ultrasound of the left axillary lymph node showing cortical thickness at the upper limit of normal

These exams revealed breast asymmetry, with the left appearing smaller and showing marked stromal densification in the upper outer quadrant. On ultrasound, this area was interpreted as a poorly defined region of diffuse heterogeneity in the echostructure. Left axillary lymph nodes were observed, with cortical thickness at the upper limit of normal (Figure 3).

An ultrasound-guided biopsy was also conducted, which revealed tissue cylinders with extensive infiltration by diffuse large B-cell NHL, non-germinal centre subtype. Neoplastic cells were CD20+, BCL2+, MUM1+, and BCL6+ in approximately 60% of the population and CD10-, CD30-, and CD5-. Nuclear expression of MYC was observed in approximately 30% of the cells (Table 1).

**Table 1:** Immunohistochemical profile of the neoplastic cells

Marker	Result
CD20	Positive
BCL2	Positive
MUM1	Positive
BCL6	Positive (~60% of cells)
CD10	Negative
CD30	Negative
CD5	Negative
MYC	Nuclear expression (~30% of cells)

**Figure 4:**
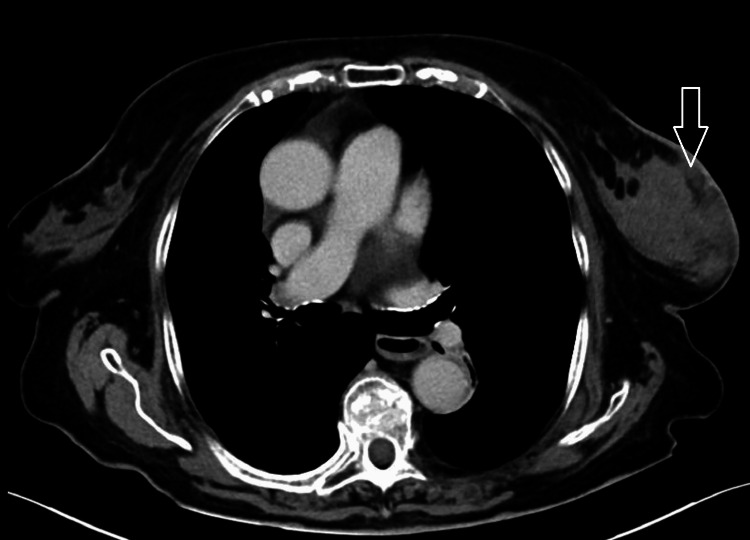
CT scan of the chest showing an infiltrative breast mass, without evidence of disease in other thoracic structures

To complete the diagnostic workup, a CT scan of the neck, chest, abdomen, and pelvis was performed, excluding disease in other locations (Figure 4). A PET-CT scan was not performed, as this is not routinely done for all patients in the public health system. 

The patient was staged as Ann Arbor IIE, with involvement of the breast and ipsilateral axillary lymph nodes. Lactate dehydrogenase (LDH) levels were within the normal range, and her Eastern Cooperative Oncology Group (ECOG) performance status was 0, reflecting full autonomy in daily activities.

The case was discussed with the haematology department, and in respect of the patient’s autonomous decision, she declined all forms of treatment, including chemotherapy and radiotherapy, and was subsequently followed by the palliative care team. She attended an initial palliative care consultation; shortly thereafter, she was hospitalised due to an infectious complication and subsequently passed away.

## Discussion

Lymphomas represent a group of malignant neoplasms that can originate in B lymphocytes, T lymphocytes, or natural killer T cells [4]. They are rare in the breast, whether as primary or secondary tumours [5]. The diagnosis is based on criteria [2,4,5] defined by Wiseman and Liao in 1972 [2], which help distinguish lymphoma from other breast diseases: the lymphoma must be localised in the breast with direct involvement of the breast tissue; a sufficient tissue sample must be available for detailed histopathological analysis; there should be no dissemination to distal lymph nodes or other organs at the time of initial diagnosis; simultaneous involvement of ipsilateral lymph nodes is acceptable, provided that the disease is confined to the ipsilateral breast and lymphatic region; extramammary lymphoma should be excluded, meaning the possibility of lymphoma originating in other organs and spreading to the breast must be ruled out [2,4,5]. Some scholars define PBLs as those originating in or confined to breast tissue, even in cases where there is distant lymph node metastasis or bone marrow involvement [5]. PBL is typically of the non-Hodgkin B-cell type [4]. The most common form of NHL is DLBCL, although it is rare as a primary breast tumour [3-6].

PBL most frequently occurs between the ages of 60 and 65 [2]. The incidence is higher in women, being very rare in men [6]. It is believed to originate from MALT, lymphoid tissue near the breast ducts and lobes, or potentially from intra-mammary lymph nodes [2]. No specific risk factors have been described; however, given the predominance in women, particularly those undergoing hormone replacement therapy, a hypothesis has been raised that estrogens may play a role in its pathophysiology. Similar to other lymphomas, immunodeficiency or immunosuppression also appears to be a risk factor [4].

The most common presentation of PBL is a painless, rapidly growing palpable nodule, with or without axillary lymphadenopathy [2,4], typically involving the outer quadrants of the breast [5,6]. It can also present as multiple lumps in some cases [2]. Skin retraction, erythema, Peau d’orange, and nipple discharge are usually not present [2,5] and occur less frequently [4]. B symptoms, such as weight loss, fever, and night sweats, are also less common in PBL [4].

The diagnosis involves the use of imaging exams, such as mammography, ultrasound, and MRI, to visualise the mass and obtain a biopsy [3]. PET-CT may also be a relevant exam for staging and monitoring lymphoma patients, as it can reveal the involvement of axillary nodes or other extranodal sites. It can also help assess treatment response [2]. However, imaging features of PBL are nonspecific [6] and do not help distinguish it from other breast lesions [5], whether malignant or benign [6]. The diagnosis is confirmed by histology [2]. The biopsy can be excisional or performed with a cutting needle (less invasive). Fine-needle aspiration is not recommended, as it compromises histopathological data and prevents lymphoma subclassification [4,6]. The study should be completed with immunohistochemistry [4].

Although clinical and imaging presentations of breast lymphoma and carcinoma are similar, their management differs significantly [6]. The treatment of PBL remains controversial, with no consensus on the best approach, which depends on histological subtype and stage [2]. Typically, it involves chemotherapy and radiotherapy [3,4]. The regimens are usually cyclophosphamide, hydroxydaunorubicin (doxorubicin), oncovin (vincristine), and prednisone (CHOP) or CHOP-like protocols [3,6]. Moreover, the addition of rituximab, an anti-CD20 monoclonal antibody, has improved outcomes. As a result, rituximab-CHOP (R-CHOP) has become first-line therapy, improving both survival and progression-free survival. For high-risk DLBCL, dose-adjusted etoposide, prednisone, vincristine, cyclophosphamide, and doxorubicin combined with rituximab (DA-EPOCH-R) is used [1]. Radiation therapy of the breast and regional lymph nodes is administered to eliminate any residual disease, improving local control and reducing the risk of recurrence [1]. In contrast to carcinoma, mastectomy is not the primary treatment option [6], as it has not shown an impact on survival [4] or recurrence risk [2], and may delay the initiation of chemotherapy [6]. However, it can be useful for diagnostic purposes when non-invasive techniques fail or in the case of a painful or haemorrhagic mass [2].

Beyond standard chemotherapy regimens, recent molecular studies have further refined our understanding of DLBCL, highlighting subtypes and genetic alterations that can influence prognosis and guide emerging therapeutic strategies. DLBCL can be classified into germinal centre B-cell-like (GCB) and activated B-cell-like (ABC) subtypes, with GCB generally associated with more favourable outcomes. Beyond these classical subtypes, additional genetic alterations define high-risk subgroups, including “double-hit” or “double-expressor” DLBCL. These molecular features not only provide prognostic information independent of clinical indices but also help guide therapeutic strategies, particularly regarding the selection of targeted agents and novel therapies in specific patient subgroups [8].

Prognosis is influenced by stage, Integrated Pulmonary Index (IPI) score, molecular subtype, and genetic rearrangements such as MYC, BCL2, or BCL6, with double-hit lymphomas associated with poorer outcomes. Five-year overall survival can range from 50% to 80%, depending on these factors [8]. Long-term multidisciplinary follow-up is essential. PBLs generally have better survival than secondary involvement [1].

This case is notable for several reasons. The patient was elderly (86 years), which is relevant, as management may need to be adapted for frail patients. She presented with an incidental, asymptomatic breast mass and declined all forms of treatment, highlighting both the challenges of patient autonomy and the need for individualised management strategies. These factors underscore real-world heterogeneity in PBL care, where outcomes may differ from published averages, and the importance of individualised management in PBL.

## Conclusions

Although rare, PBL should be considered in the differential diagnosis of breast masses. It is an aggressive tumour with high relapse rates and generally worse prognosis compared to breast carcinoma, requiring a distinct treatment strategy. Timely and accurate diagnosis followed by appropriate therapy is crucial, and ongoing follow-up is essential due to the risk of recurrence. A multidisciplinary approach is key to optimise management, improve prognosis, and avoid unnecessary mastectomies. Importantly, patient-centred, individualised decisions are essential, particularly in frail elderly patients, to ensure treatment aligns with patient preferences and overall health status.
